# Antimicrobial Activity of Necklace Orchids is Phylogenetically Clustered and can be Predicted With a Biological Response Method

**DOI:** 10.3389/fphar.2020.586345

**Published:** 2021-03-12

**Authors:** Richa Kusuma Wati, Esmée F. de Graaf, Diego Bogarín, Reinout Heijungs, Rogier van Vugt, Erik F. Smets, Barbara Gravendeel

**Affiliations:** ^1^ Naturalis Biodiversity Center, Endless Forms Group, Leiden, Netherlands; ^2^ Center for Plant Conservation, Bogor Botanic Garden, Indonesian Institute of Sciences (LIPI), Bogor, Indonesia; ^3^ Science and Technology Faculty, University of Applied Sciences Leiden, Leiden, Netherlands; ^4^ Lankester Botanical Garden, University of Costa Rica, Cartago, Costa Rica; ^5^ Institute of Environmental Sciences, Leiden University, Leiden, Netherlands; ^6^ Department of Econometrics and Operations Research, Vrije Universiteit Amsterdam, Amsterdam, Netherlands; ^7^ Hortus botanicus, Leiden University, Leiden, Netherlands; ^8^ Institute of Biology Leiden, Leiden University, Leiden, Netherlands; ^9^ Ecology, Evolution and Biodiversity Conservation, KU Leuven, Heverlee, Belgium; ^10^ Institute of Water and Wetland Research, Radboud University, Nijmegen, Netherlands

**Keywords:** bio-assays, bioprospecting, Coelogyninae, herbal medicine, horticulture, hot nodes

## Abstract

Necklace orchids (Coelogyninae, Epidendroideae) have been used in traditional medicine practices for centuries. Previous studies on a subset of unrelated orchid species utilized in these traditional practices revealed they possessed antimicrobial, anti-inflammatory, and anti-oxidant activity, providing experimental proof for their medicinal properties. To date however none of these species have been investigated ethno-botanically in a phylogenetic context. This study carried out comparative bioprospecting for a group of wild orchids using EBDCS (the Economic Botany Data Collection Standards) organ targeted and biological response methods. The traditional medicinal use of necklace orchids was recorded from books and journals published between 1984 and 2016. Two orchids, *Coelogyne cristata* and *Coelogyne fimbriata*, were selected, cultivated both indoors and outdoors, and the antimicrobial properties on extracts from their leaves and pseudobulbs tested against a selection of human pathogens. A molecular phylogeny of Coelogyninae based on nuclear ribosomal ITS and plastid *matK* DNA sequences obtained from 148 species was reconstructed with Maximum Likelihood (ML) using RAxML, Maximum Parsimony (MP) using PAUP and Bayesian Inference using MrBayes. Bioprospecting comparison of EBDCS and biological response was carried out using customized R scripts. Ethanolic extracts obtained from leaves of *C. fimbriata* inhibited growth of *Bacillus cereus*, *Staphylococcus aureus*, and *Yersinia enterocolitica,* confirming the antimicrobial properties of these extracts. Leaf extracts were found to have slightly stronger antimicrobial properties for plants cultivated outdoors than indoors. These differences were not found to be statistically significant though. Three hot nodes with high potency for antimicrobial activities were detected with the EBDCS organ targeted classification method, and eight hot nodes were detected with the biological response classification method. The biological response classification method is thus a more effective tool in finding hot nodes amongst clades of species with high medicinal potential.

## Introduction

For millennia, products of nature have been an important source of traditional medicine (Cragg and Newman, 2013). Even today, between 70 and 95% of the world population in developing countries continues to use traditional medicines (Robinson and Zhang, 2011). Plant-based antibiotics form the basis of these traditional medicinal systems (Newman et al., 2000). There is an increasing interest in the study of these plant-based antibiotics as a source of novel antibiotics that human pathogens may not have developed resistance against, and because of the increasing extinction rate of wild plant species (Savoia, 2012; Cragg and Newman, 2013; Ernst et al., 2016).

To discover potential new plant species with antimicrobial properties, a time-efficient and systematic approach is needed. Bioprospecting is an approach combining phylogeny with ethnobotanical knowledge to identify potential sources of bioactive compounds. The underlying assumption is that phylogenies can predict the traditional medicinal use of natural products in a bioprospecting approach (Saslis-Lagoudakis et al., 2012; Leonti et al., 2013; Ernst et al., 2016). The hypothesis is that closely related species share similar biochemical pathways and that the same bioactive compounds are present in all descendants of a single ancestor rather than in species scattered over unrelated clades. This method has been used in different plant species (Douwes et al., 2008; Zhu et al., 2011; Saslis-Lagoudakis et al., 2012; Siqueira et al., 2012; Leonti et al., 2013) and animal groups (Smith and Wheeler, 2006). For bioprospecting, two different methods are mainly used. The first method is the Economic Botany Data Collection Standard (EBDCS) classification method. The EBDCS provides a system where cultural plant uses are described using standardized descriptors and terms, and attached to taxonomic data sets. This classification is based on the treatment of symptoms, i.e. a medicine against stomach pain (Cook, 1995). The other method is a classification based on the biological response, such as a medicine with antimicrobial effects (Ernst et al., 2016).

Pathogens cause an array of diseases in humans, and their identification is important in administering the correct treatment. (Washington, 1996). It is expected that bioprospecting based on biological responses will produce different results from the organ targeted EBDCS method, as biological responses are focused on a classification based on a single effect in the entire human body rather than a single organ (Ernst et al., 2016). A growing number of studies report on the bioprospecting of medicinal plants, including orchids (Beena, 2011; Purkayastha, 2016). We have not yet come across any study carried out on a group of wild orchids from a phylogenetic perspective.

**FIGURE 1:**
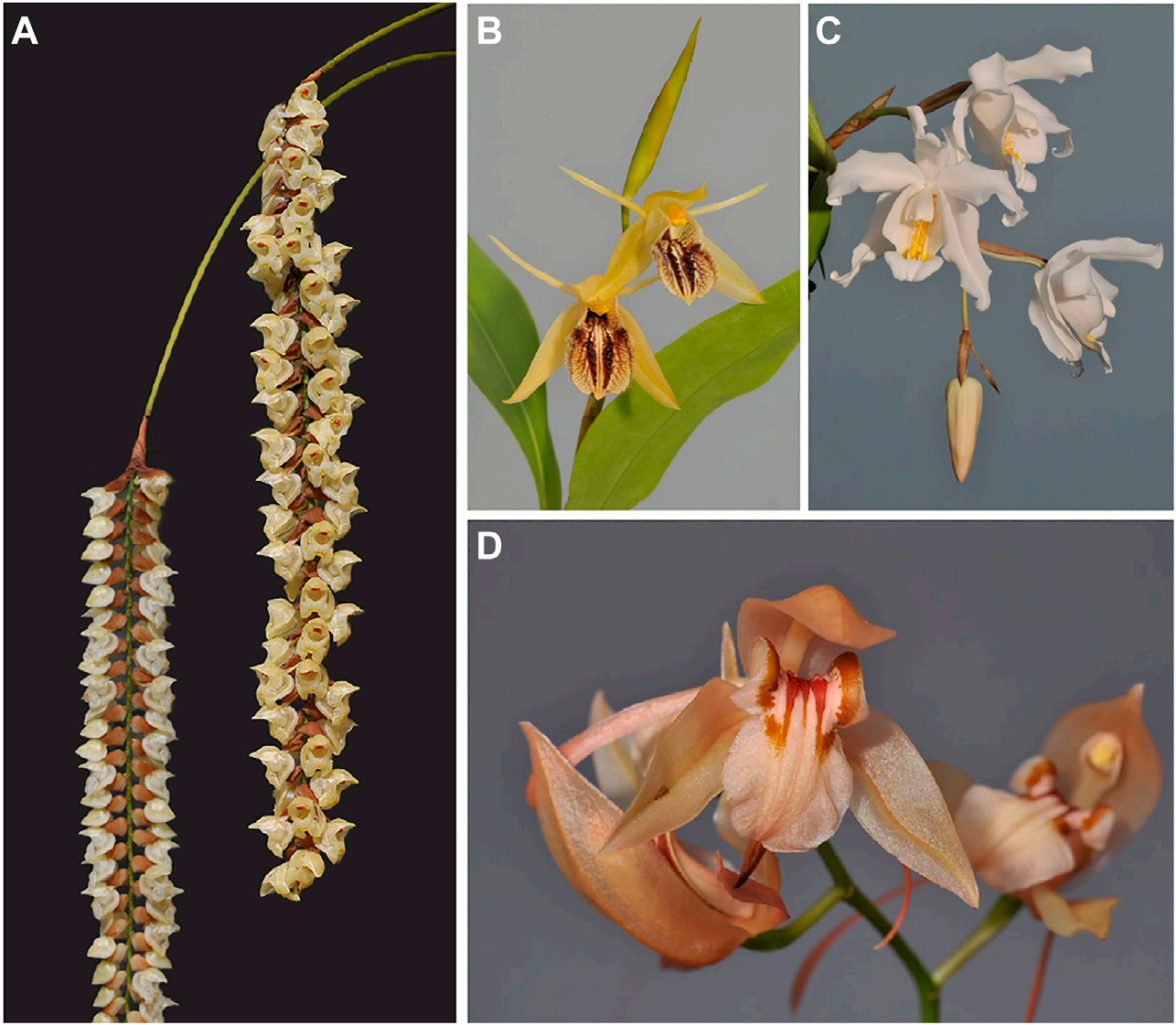
Examples of medicinally used necklace orchids investigated in this study. **(A)** *Pholidota imbricata.* **(B)** *Coelogyne fimbriata*. **(C)** *Coelogyne cristata*. **(D)** *Coelogyne fuscescens*. The *Pholidota* species depicted under A can only be cultivated in a humid greenhouse. The *Coelogyne species* depicted under B-D can be cultivated outdoors during the summer and early fall in temperate regions to stimulate the production of secondary compounds. Photographs by Rogier van Vugt.

The orchid family is historically well-known for its medicinal properties (Lawler, 1984; Singh and Singh, 2012). Medicinal orchids contain phytochemicals such as alkaloids, bibenzyl derivatives, flavonoids, phenanthrenes and terpenoids, which are present in leaves, roots, pseudobulbs (modified stem parts for water and nutrient storage), and flowers (Gutiérrez, 2010; Hsiao et al., 2011; Pant, 2014). Necklace orchids (Coelogyninae, Epidendroideae) comprise over 680 species, that are distributed throughout Southeast Asia (Pridgeon et al., 2005). *Bletilla, Coelogyne, Dendrochilum, Otochilus, Pholidota*, *Pleione*, and *Thunia* are examples of necklace orchid genera with documented medicinal properties (Singh and Duggal, 2009; Subedi et al., 2011; Pant and Raskoti, 2013; Teoh, 2016) (see Figure 1).

In this study, we 1) compiled traditional medicinal uses of necklace orchids from the literature, 2) carried out bio-assays on six human pathogens with ethanol and hexane extracts of leaves and pseudobulbs from *C. cristata* and *C. fimbriata* plants grown both inside a glasshouse and outside to experimentally validate whether traditional growth methods impacted the orchid medicinal properties, and 3) investigated whether an organ-targeted EBDCS or biological response-based classification was most informative for predicting the biological activity of related species.

## Materials and Methods

### Medicinal Uses of Necklace Orchids Recorded in the Literature

Information on the medicinal use of different species of necklace orchids was compiled from scientific journals and books through September 2019 (Table 1). We included all data from publications that stated the local names, latin names and the traditional uses for the orchid species. We excluded the publications where only the local name and/or genus were given. All records were compiled into a list and coded according to the Economic Botany Data Collection Standard (EBDCS) as recommended by the Biodiversity Information Standards of the Taxonomic Databases Working Group (TDWG) (Cook, 1995). The medicinal properties of the orchid species were categorized into EBDCS level 2 characters and into biological response characters. We used the antimicrobial response character as defined in MedlinePlus with three different states: no response, possible response or unknown response. This definition assumes that a plant species should be categorized as no response when it is applied for anything other than antimicrobial treatments, such as bone fracture treatments. A possible response was scored when the disease dictionary of MedlinePlus dictated this. Reducing fever was for instance scored as a possible antimicrobial effect since fever is a biological response to infection. Finally, an ‘unknown response’ was given if no records of plant use were available. Medicinal properties of all *Glomera* species were categorized as unknown, as to the best of our knowledge no ethnobotanical information for this genus has been published.

**TABLE 1:** Information on traditional medicinal use of necklace orchids (Coelogyninae) compiled from the literature.

Species	Use		References
	Symptoms	Plant organ(s)	
*Bletilla formosana* (Hayata) Schltr.	Strengthen the lungs, stop bleeding and reduce swellings. Used for treatment of tuberculous cough, bronchiectasis, bleeding peptic ulcers, nose-bleed and treat cracks on the heel	Stems	Teoh (2016)
*Bletilla ochracea* Schltr.	See *B* *. striata*	Tubers/pseudobulbs	Teoh (2016)
*Bletilla foliosa* (King & Pantl.) Tang & F.T.Wang	See *B* *. striata*	Tubers/pseudobulbs	Teoh (2016)
*Bletilla striata* (Thunb.) Rchb.f.	Benefit the lungs (effect on pulmonary diseases), liver and stomach meridians. Effects of the medicine are haemostatic, reduce swelling and promotes regeneration of muscles and other tissues. Also used to treat sores, pustules and dry, chapped and burned skin	Tubers/pseudobulbs	Teoh (2016)
*Coelogyne barbata* Lindl. Ex Griff.	The whole plant is valued for its ability to counter 'heat', relieve thirst, stop coughs and lessen pain. It is used to treat sore throat, pain at hernias, swelling of the scrotum, chappy extremities, traumatic injuries and 'lung-heat'	Entire plant	Teoh (2016)
*Coelogyne corymbose* Lindl.	Paste applied to the forehead to relieve headaches, fresh juice applied to burns and wounds as an analgesic. It treats fractures and is used as haemostatic and to relieve pain. Reduces heat and taken for coughs, flu, and bronchitis	Pseudobulbs/entire plant	Pant and Raskoti (2013), Subedi et al. (2011, 2013), Teoh (2016), Vaidya et al. (2000), Yonzone et al. (2012)
*Coelogyne cristata* Lindl.	Are given for constipation as well as diarrhoea and dysentery. It is also used as an aphrodisiac. Freshly collected paste or juice consumed to relieve headaches, fever and for indigestion. Pulp applied to burnt skin. Juice also applied to wounds and skin boils. Gum is used for sores. Used for cooling & soothing	Pseudobulbs	Pant and Raskoti (2013), Subedi et al. (2011, 2013), Teoh (2016), Vaidya et al. (2000)
*Coelogyne fimbriata* Lindl.	Powder used in tonic preparation and used to reduce heat	Pseudobulbs	Subedi et al. (2011, 2013), Teoh (2016)
*Coelogyne flaccida* Lindl.	Paste applied externally or consumed to relieve frontal headaches, fever, and boils. Juice is taken for indigestion. The whole plant is also used to clear heat, counter dryness, promote the production of body fluids, clear phlegm and stop coughs	Pseudobulbs/entire plant	Pant and Raskoti (2013), Teoh (2016)
*Coelogyne fuscescens* Lindl.	Paste applied externally or consumed to relieve headaches, fever, and stomach/abdominal ache. Treat burns and otitis media. Has sometimes an aphrodisiac function	Pseudobulbs	Pant and Raskoti (2013), Subedi et al. (2011), Yonzone et al. (2012, 2013), Teoh (2016), Vaidya et al. (2000)
*Coelogyne nitida* (Wall. Ex D. Don) Lindl.	Juice consumed against headaches and fever and recommended for stomach ache. Paste applied externally on burns	Pseudobulbs	Pant and Raskoti (2013), Subedi et al. (2011, 2013), Teoh (2016)
*Coelogyne ovalis* Lindl.	Used as a tonic, aphrodisiac and to treat coughs, urine infections and eye disorders	Not specified	Pant and Raskoti (2013), Teoh (2016), Yonzone et al. (2012, 2013)
*Coelogyne prolifera* Lindl.	Paste consumed against headaches and fever. Paste applied externally on burns, boils and to relieve backache	Pseudobulbs	Pant and Raskoti (2013), Subedi et al. (2011, 2013), Teoh (2016)
*Coelogyne punctulata* Lindl.	Used to treat wounds, burns, dry coughs. Relieves pain and helps to heal the wounds	Pseudobulbs	Teoh (2016), Yonzone et al. (2012, 2013)
*Coelogyne stricta* (D.Don) Schltr.	Paste applied externally against headaches and fever. Healing of fractured bones	Pseudobulbs	Pant and Raskoti (2013), Subedi et al. (2011, 2013), Teoh (2016), Vaidya et al. (2000), Yonzone et al. (2013)
*Coelogyne trinervis* Lindl.	Used to treat fractures and sprains	Tuber	Teoh (2016)
*Otochilus lancilabius* Seidenf.	Paste applied to fractured and dislocated bones	Entire plant	Subedi et al. (2011, 2013)
*Pholidota articulata* Lindl.	Paste applied on fractured bones and consumed as a tonic. Root powder is used to treat cancer. Juice berries are used to treat ulcers, skin eruptions, traumatic injuries, and sores. Removes gas and reduce swelling. Also used to treat coughs caused by body heat, headache, dizziness, irregular menses, and uterine prolapse	Entire plant	Pant and Raskoti (2013), Subedi et al. (2011, 2013), Teoh (2016), Vaidya et al. (2000)
*Pholidota cantonensis* Rolfe	Used to treat high fever, eczema, and haemorrhoids	Entire plant	Teoh (2016)
*Pholidota chinensis* Lindl.	Used for cooling, moistens the lungs, promotes salivation. Used to treat tuberculosis-associated haemoptysis, acute or chronic bronchitis, dry cough, pharynchitis, tonsillitis, toothache, peptic ulcer, gastroenteritis, dizziness, headache, post-concussion syndrome, neurasthenia, osteomyelitis and trauma	Entire plant	Teoh (2016), Yonzone et al. (2012), Wang et al. (2006)
*Pholidota imbricata* Hook.	Paste consumed to relieve fever and powder as a tonic. Juice is applied to relieve navel pain, abdominal pain, rheumatic pain, and headache. Applied to boils and to treat fractures	Pseudobulbs	Pant and Raskoti (2013), Subedi et al. (2011, 2013), Teoh (2016), Vaidya et al. (2000), Yonzone et al. (2012, 2013)
*Pholidota pallida* Lindl.	Paste used to relieve fever, powder to induce sleep and to cure abdominal pain, juice used for navel pain and rheumatic pain and sore throat	Rhizome, pseudobulbs	Pant and Raskoti (2013), Subedi et al. (2011, 2013), Teoh (2016), Vaidya et al. (2000), Yonzone et al. (2012, 2013)
*Pleione bulbocodioides* (Franch.) Rolfe	Treatment for wet sores, sore throat, rabies, tuberculosis, asthma, boils, and carbuncles. Clears phlegm. It reduces inflammation and fever. It removes extravasated blood swellings. It is also used as a detoxifier	Entire plant	Teoh (2016)
*Pleione hookeriana* (Lindl.) Rollisson	Are used to remove heat, toxins, abscesses and lymphatic tuberculosis	Pseudobulbs	Teoh (2016)
*Pleione humilis* (Sm.) D.Don	Paste applied on cuts and wounds. Powder used as a tonic	Pseudobulbs	Pant and Raskoti (2013), Subedi et al. (2011, 2013), Teoh (2016)
*Pleione maculata* (Lindl.) Lindl. & Paxton	Used for liver and stomach ailments	Pseudobulbs	Pant and Raskoti (2013), Subedi et al. (2013), Teoh (2016), Vaidya et al. (2000), Yonzone et al. (2012, 2013)
*Pleione praecox* (Sm.) D.Don	Dried powder consumed (with milk) as tonic and energizer. Paste externally applied on cuts and wounds	Pseudobulbs	Pant and Raskoti (2013), Subedi et al. (2011, 2013), Teoh (2016)
*Thunia alba* (Lindl.) Rchb.f.	Paste used on fractured and dislocated bones. Benefit the lungs, clear phlegm and stop cough, remove bruises and improve blood flow	Entire plant	Pant and Raskoti (2013), Subedi et al. (2011, 2013), Teoh (2016)

### Antimicrobial Activity

#### Plant Material

Fresh pseudobulbs and leaves of mature sterile plants of *C. cristata* and *C. fimbriata* (3–5 different individuals per species) grown in greenhouses were obtained from Orchideeën Wubben (Hollandsche Rading, Netherlands) and Claessen Orchideeën (Nederweert-Eind, Netherlands). The same species were subsequently grown outside where they were exposed to UV light and herbivorous snails and insects during a period of 5 months in the Hortus botanicus (Leiden, Netherlands). A second batch of fresh pseudobulbs and leaves was then harvested from these species. All leaves and pseudobulbs were sterilized and freeze-dried in a VirTis Benchtop Pro Freeze Dryer at −104°C until they reached a constant weight. The dried pseudobulbs and leaves were ground into a fine powder and about 1 g of the powder was extracted with 70% ethanol and 100% hexane in a vacuum speed extractor E-916 (Buchi, Switzerland) (40°C, 100 bar). The extracts were stored in a freezer (−20°C) before further use.

#### Bacterial Strains

The antimicrobial properties of extracts from pseudobulbs and leaves of *C. cristata* and *C. fimbriata* were evaluated with five bacterial strains that are common causes of human gastrointestinal tract infections and are resistant against a range of synthetic antibiotics (Mutsaers et al., 2001). Two strains of Gram-positive *Staphylococcus aureus* (ATCC 12600) and *Bacillus cereus* (ATCC 14579) and three strains of Gram-negative *Escherichia coli* (ATCC 10798)*, Klebsiella pneumoniae* (ATCC BAA-3079)*,* and *Yersinia enterocolitica* (ATCC 9610) bacteria were used for the experiments. The bacterial strains were provided by the University of Applied Sciences Leiden, Netherlands. All strains were cultured on Columbia Agar with 5% Sheep Blood (COL-S (BDTM)) overnight at aerobic conditions at 37°C (except for *Y. enterocolitica*, which was typically cultured at 30°C), followed by storage at 4°C for up to 1 week.

#### Antimicrobial Activity of Plants Extracts

A disk diffusion method was used to evaluate the antimicrobial activity for each plant extract. Each bacterial strain was streaked onto a plate, grown overnight, and used to inoculate Mueller-Hinton cation-adjusted agar broth 2 (Sigma-Aldrich). The cultures were incubated overnight under aerobic conditions at 37°C (except for *Y. enterocolitica*, which was grown at 30°C) on a rotary shaker (180 rpm) until a McFarland Standard of 0.5 (10^7^ CFU/ml) was reached. The cultures were subsequently used to make a confluent growth on COL-S agar plates. Sterile filter paper disks with (10 mm diameter, Sigma-Aldrich) were loaded with the different plant extracts with a total content of 55 mg/ml. The disks were then evaporated by air at room temperature inside a laminar air flow hood for 20 min before they were placed onto the top of the inoculated plates. Sterile filter paper disks loaded with 7.5 µg of Levofloxacin (Sigma-Aldrich) were used as positive control, and sterile paper disks loaded with 5% DMSO (Sigma-Aldrich) were used as the negative control. All the samples were then incubated at 37°C (except for *Y. enterocolitica* at 30°C) for 24 h. All tests were performed in triplicate and the zones of inhibition were measured with an automatic Vernier caliper. The scale of the inhibitory effect was scored as follows: high (diameter zone ≥17 mm), intermediate (14 ≤ diameter zone <16 mm), low (diameter zone ≤13 mm) (CLSI, 2011).

### Phylogenetic Reconstructions

#### Plant Sampling and DNA Extraction

Previously generated DNA sequences for necklace orchids (Gravendeel et al., 2001; Subedi et al., 2011; Sulistyo et al., 2015; Pedersen et al., 2020) were downloaded from NCBI GenBank (see Supplementary Table S2 for more details). In addition, new DNA sequences were generated from 77 specimens of the necklace orchid genus *Glomera*. From these, 14 specimens were collected in the field in Seram, Papua and Papua New Guinea (Indonesia). The first author also identified living orchid material from the Bogor Botanical Garden with identification keys and taxonomic descriptions from Schuiteman and de Vogel (2001), Wati et al. (2018) and the website of de Vogel et al. (2019). Additionally, a total of 42 specimens from the living orchid collection of the Hortus botanicus Leiden, Netherlands were analysed. Lastly, 21 dried herbarium specimens from the Herbarium Bogoriense, Indonesia and the herbarium of Naturalis Biodiversity Center, Leiden, Netherlands, were analysed (see Supplementary Table S2 for more details). Total genomic DNA was extracted from 50 mg of leaf tissue from herbarium or silica-gel dried material using the 2x CTAB (Cetyltrimethylammonium bromide) method of Doyle and Doyle (1987), or with the Qiagen DNeasy Plant mini kit (Qiagen) following the manufacturer’s protocol.

#### Amplification and Sanger Sequencing

The nuclear ribosomal ITS-5.8S-ITS2 (nrITS) region of silica-gel dried leaf material was amplified using primers 17SE (5′-ACG​AAT​TCA​TGG​TCC​GGT​GAA​GTG​TTC-3′) and 26SE (5′-TAG​AAT​TCC​CCG​GTT​CGC​TCG​CCG​TTA​C-3′) as described by Sulistyo et al. (2015). Subsequently, a M13 universal sequencing primer was added to the 5′ end of the forward (ACG​AAT​TCA​TGG​TCC​GGT​GAA​GTG​TTC) and reverse (TAG​AAT​TCC​CCG​GTT​CGC​TCG​CCG​TTA​C) primers to improve Sanger sequencing efficiency. Each PCR reaction was 25 µL and included the template DNA, CoralLoad PCR buffer (Qiagen), dNTPs, Taq DNA Polymerase (Qiagen), and both primers. All PCR reactions were done on a C1000 Touch Thermal Cycler (Bio-Rad) instrument. The thermal cycling protocol began with a 5 min initial denaturation at 96°C, followed by 35 amplification cycles, each with 30 s denaturation at 96°C, 30 s annealing at 50°C, and 1 min extension at 72°C, followed by a final 7 min final extension at 72 °C.

The nrITS region of herbarium preserved leaf material was amplified using primer p3 (5′-GACTCYCGGCAATGGATATCTCG-3′) and p4 (5′-CCGCTTATTGATATGCTTAAACTCRGC-3′) as described by Cheng et al. (2016) and primer efgF1 (5′-CGA​GTC​TTT​GAA​CGC​AAG​TTG​CG-3′) and efgR1 (5′-GGC​CAA​CGA​GAC​GAT​AAC​CC-3′) that were newly designed. Each PCR reaction consisted of 25 μL, containing the template DNA, 5x Phire PCR buffer (ThermoScientific), BSA, dNTPs, Phire Hot Start II DNA Polymerase (ThermoScientific), and both primers. The thermal cycling protocol began with a 1 min initial denaturation at 98°C, followed by 40 amplification cycles, each with 10 s denaturation at 98°C, 10 s annealing at 50°C, and 20 s extension at 72°C, followed by a 1 min final extension at 72 °C.

The *matK* region of silica dried silica-gel dried leaf material was amplified using two primer sets: 731F (5′-TCT​GGA​GTC​TTT​CTT​GAG​CGA-3′) and 2R (5′-AAC​TAG​TCG​GAG​TAG-3′), and 19F (5′-CGT​TCT​GAC​CAT​ATT​GCA​CTA​TG-3′) and 881R (5′-TMTTCATCAGAATAAGAGT-3′) as described by Sulistyo et al. (2015). The PCR reaction setup was the same as for nrITS with fresh plant material, but with additional BSA. The thermal cycling protocol began with a 5 min initial denaturation at 94°C, followed by 35 amplification cycles, each with a 1 min denaturation at 94°C, 30 s annealing at 50°C, and 1 min extension at 72°C, followed by a 7 min final extension at 72°C.

Sanger sequencing of the amplification products were performed at Baseclear (http://www.baseclear.com/), using an ABI-3730XL DNA Sequencer (Applied Biosystems). All sequences were deposited in NCBI GenBank. Accession numbers of all sequences can be found in Supplementary Table S2.

#### Sequence Editing and Phylogenetic Analysis

Sanger sequences were assembled and edited in Geneious^®^ R8 (Biomatters Ltd., Auckland, New Zealand) (Kearse et al., 2012). The ends of all data sets were trimmed to avoid character misinterpretation. Ambiguous bases were replaced with “N” in the data matrix. DNA sequences were aligned using the MAFFT platform (Multiple Alignment Fast Fourier Transform) (Katoh and Standley, 2013) as implemented in Geneious^®^ R8 with subsequent manual adjustment. Missing data were replaced with “?”.

A phylogenetic analysis was carried out using Bayesian Inference (BI) with *Arundina graminifolia* as an outgroup based on earlier studies (Gravendeel et al., 2001; Pedersen et al., 2020) that showed this genus to be most closely related to the necklace orchids. The chosen nucleotide substitution model GTR+G was calculated using the Akaike Information Criterion (AIC) in jModelTest2 v.2.1.6 (Darriba et al., 2015). The analyses were run in the CIPRES Science Gateway v.3.1. (Miller et al., 2010). We performed Bayesian interference analyses with Mr. Bayes v.3.2.6 on XSEDE (Huelsenbeck et al., 2004) with the following parameters for the alignment dataset: number of runs (nruns = 2), number of chains to run (nchains = 4), number of generations (ngen = 5 × 10^7^), temperature parameter (temp = 2) and sampling frequency of 2000 yielding 25,000 trees per run. The log files from MrBayes were inspected in Tracer v.1.6 to check for convergence of independent runs (i.e. with estimated sample size (ESS) > 200). Maximum Likelihood analyses were performed with RAxML-HPC2 on XSEDE (8.2.10) (Stamakis et al., 2008) choosing the GTRGAMMA model for bootstrapping and 1,000 bootstrap iterations. Parsimony analyses were performed with PAUPRat: Parsimony ratchet searches using PAUP* (Nixon, 1999; Sikes and Lewis, 2001; Swofford, 2002) with 1,000 ratchet repetitions, seed value = 0, 20% percent of characters to perturb (pct = 20), original weights 1 for all characters (wtmode = uniform) and a tree bisection-reconnection branch swapping algorithm (swap = TBR). The 50% majority rule consensus for MP was obtained with PAUP v4.0a152. and inspected in FigTree v.1.3.1. The statistical support of the clades was evaluated with the values of posterior probability (PP) for BI reconstruction, bootstrap for ML (MLB) and parsimony bootstrap for MP (MPB). The support values (PP) were added to the branches on the Bayesian 50% majority-rule consensus tree with additional support values shown for ML and MP when the same topology was retrieved.

### Bioprospecting Analysis

A randomly selected subset of 1.000 trees within the 95% highest posterior density (HPD) interval was used for further analyses using the packages *caper, ape*, *plyr*, and (Paradis et al., 2004; Kembel et al., 2010; Wickham, 2011; Orme et al., 2013) scripts in the R programming language (R Core Team, 2018) under RStudio (Gandrud, 2015). The R bioprospecting script of Ernst et al. (2016) was used to assess evolutionary patterns of medicinal properties of the necklace orchids analysed. The strength of the phylogenetic signal of the EBDCS category and the antimicrobial biological response category were investigated using D statistics (Fritz and Purvis, 2010), that was calculated with the *phylo. d* function implemented in the R package *caper* (Orme et al., 2013). A boxplot of the D values for each category of the two classification methods investigated was made using *ggplot*. If 95% of the 1.000 trees had a median value of D > 1, the medicinal properties were considered as randomly distributed; for D < 1, the phylogenetic signal was considered as strong (Ernst et al., 2016). D > 0 indicates that the medicinal properties of the orchids possess a significantly different distribution from the standard Brownian model, implying that they are clustered within the phylogeny. D < 0 indicates that the categories are extremely clustered. The prevalence of each category was measured by N_total species included in the category_/N_total number of species_. For a prevalence <0.020 the category was considered as too biased, and omitted from further analyses.

We also tested the phylogenetic diversity (PD) of the EBDCS category and the antimicrobial biological response category with the function *pd* in the R package *picante* v.1.6-2 (Kembel et al., 2010). The percentage of the possible response category of the antimicrobial biological response was compared with the Infections/Infestations category of the EBDCS classification method. A higher PD percentage means that species in this category are more scattered throughout the phylogeny. As a consequence, more potential species with medicinal properties are present because the PD-values are based on the total branch length spanned by the species (Ernst et al., 2016).

A consensus BI tree with 10% burnin was used to visualize the distribution of the two categories over the necklace orchid species investigated. Using the *nodesigl* command in R with the system PHYLOCOM v4.2 (Webb et al., 2008), so-called ‘hot nodes’ were calculated to visualize potential medicinal species.

## Results

### Medicinal Uses of Necklace Orchids Recorded in the Literature

For 28 necklace orchid species, traditional medicinal uses were compiled to determine 19 organ-targeted categories and a single biological response (i.e., antimicrobial) category with three different character states (see Tables 1, 2, and 3 for an overview of all data obtained from the literature). The prevalence of the categories Mental Disorders, Nervous System Disorders and Sensory System Disorders in the EBDCS classification method showed the lowest value of 0.006 because only one species was used in these categories.

**TABLE 2:** Prevalence of various categories of medicinal use of necklace orchids for the organ targeted EBDCS classification method.

EBDCS classification method	N_Total species included in the category_	Prevalence
Abnormalities	5	0.033
Circulatory system disorders	2	0.013
Digestive system disorders	14	0.094
Genitourinary system disorders	5	0.033
III-defined symptoms	2	0.013
Infections/infestations	13	0.087
Inflammations	3	0.020
Injuries	16	0.108
Mental disorders	1	0.006
Metabolic system disorders	9	0.060
Muscular-skeletal system disorders	13	0.087
Nervous system disorders	1	0.006
Nutritional disorders	7	0.047
Pain	12	0.081
Poisonings	2	0.013
Respiratory system disorders	13	0.087
Sensory system disorders	1	0.006
Skin/subcutaneous cellular tissue disorder	13	0.087
Unknown	120	0.810
N_Total_ (total number of species)	148	

**TABLE 3:** Prevalence of various categories of medicinal use of necklace orchids for the antimicrobial response classification method.

Antimicrobial response classification method	N_Total number of species included in the category_	Prevalence
No documented response	111	0,75
Possible response	19	0,123
Unknown process	122	0,824
N_Total_ (total number of species)	148	

### Bioassays

None of the 100% hexane leaf and pseudobulb extracts and 70% of the ethanol pseudobulb extracts showed any antimicrobial effect in the bio-assays conducted. On the contrary, the 70% ethanol leaf extracts inhibited the growth of several of the human pathogens investigated (Table 4). Extracts obtained from freshly harvested leaves of *C. cristata* and *C. fimbriata* were found to inhibit growth of *Y. enterocolitica, B. cereus* and *S. aureus* and confirmed the traditional medicine uses recorded in the literature (Pyakurel and Gurung, 2008; Subedi, 2002; Subedi et al., 2013).

**TABLE 4:** Antimicrobial activity of extracts of *Coelogyne cristata* and *C. fimbriata* as recorded in the bioassays carried out in this study of five plants per species grown in greenhouses.

Extracts	Zone of inhibition (mm)				
	*Bacillus cereus*	*Escherichia coli*	*Klebsiella pneumoniae*	*Staphylococcus aureus*	*Yersinia enterocollitica*
Positive control (7.5 μg/ml levofloxacin)	22.87 ± 1.0	13.12 ± 0.2	22 ± 2.2	14.6 ± 0.6	38 ± 1.5
70% EtOH pseudobulbs *C. cristata*	—	—	—	—	—
70% EtOH leaves *C. cristata*	—	—	—	—	—
Hexane pseudobulbs *C. cristata*	—	—	—	—	—
Hexane leaves *C. cristata*	—	—	—	—	—
70% EtOH pseudobulbs *C. fimbriata*	—	—	—	—	—
70% EtOH leaves *C. fimbriata*	15.55 ± 0.6	13.88 ± 0.7	18.55 ± 0.6	13.3 ± 1.0	21.7 ± 2.0
Hexane pseudobulbs *C. fimbriata*	—	—	—	—	—
Hexane leaves *C. fimbriata*	—	—	—	—	—

All experiments were carried out in triplicate. Absence of growth inhibition is indicated with —

The highest effect was recorded for the 70% EtOH leaf extracts of *C. fimbriata* against *Y. enterocollitica* (19.6 ± 4.2 mm). Intermediate effects were recorded for leaf extracts of the same *Coelogyne* species against *B. cereus* (14.3 ± 1.4 mm) and *S. aureus* (13.6 ± 1.2 mm). Leaf extracts were found to have slightly stronger (but not significant) antimicrobial properties for plants cultivated outdoors than indoors (Table 5).

**TABLE 5:** Antimicrobial activity of extracts of *Coelogyne cristata* and *C. fimbriata* as recorded in the bioassays carried out in this study of five plants per species grown outside for 5 months in the Hortus botanicus Leiden, Netherlands .

Extracts	Zone of inhibition (mm)				
	*Bacillus cereus*	*Escherichia coli*	*Klebsiella pneumoniae*	*Staphylococcus aureus*	*Yersinia enterocollitica*
Positive control (7.5 μg/ml levofloxacin)	22.51 ± 0.8	18.77 ± 0.4	26 ± 1.2	20.15 ± 0.2	39 ± 2.0
70% EtOH pseudobulbs *C. cristata*	—	—	—	—	—
70% EtOH leaves *C. cristata*	—	—	—	—	—
70% EtOH pseudobulbs *C. fimbriata*	—	—	—	—	—
70% EtOH leaves *C. fimbriata*	16.44 ± 0.8	14.55 ± 1.4	17.22 ± 1.0	22.55 ± 1.4	20.44 ± 1.1

All experiments were carried out in triplicate. Absence of inhibition zone is indicated with —.

### Bioprospecting of Necklace Orchids

**FIGURE 2:**
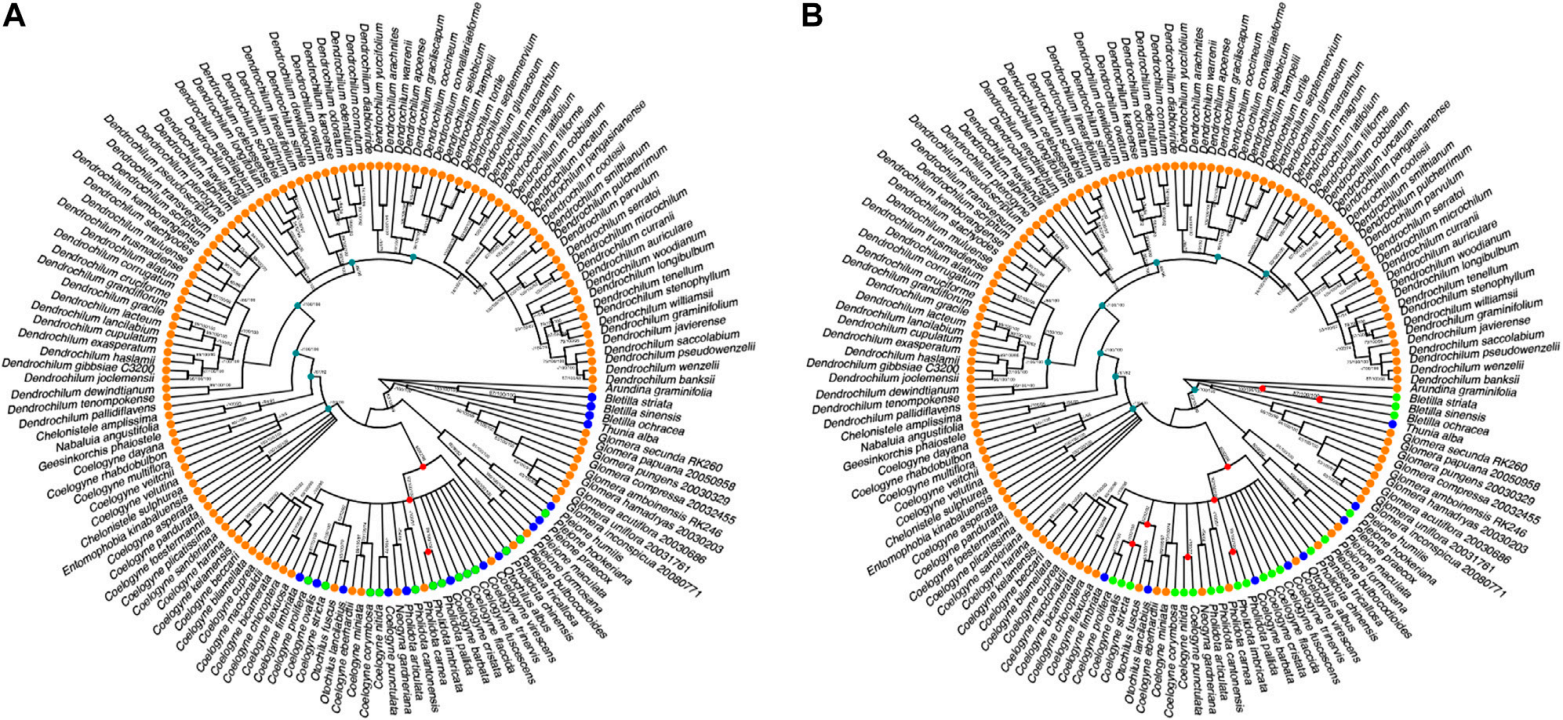
Majority consensus Bayesian Inference tree reconstructed on combined nrITS and plastid *matK* sequences of species of necklace orchids (Coelogyninae). **(A)** Plotting of the Infections/Infestation category of the organ targeted EBDCS classification method on this BI tree. **(B)** Plotting of the Antimicrobial biological response classification method. Explanation of color codes: species with no antimicrobial use (blue), species with unknown antimicrobial use (orange), species with possible antimicrobial use (light green), ancestral hot nodes of clades with high potency of species with antimicrobial properties (red). Hot nodes were identified with the *nodesigl* command in the PHYLOCOM package. Plotted branch values for Maximum Likelihood Bootstrap Support (BS), Maximum Parsimony BS, and Posterior Probability are given for each well-supported clade.

The majority consensus reconstructed BI tree, which is based on combined nrITS and plastid *mat*K sequences for 148 species of necklace orchid species, is depicted in Figure 2. The consensus tree of ML and MP shows relatively high support for (>70%) and was congruent with the topology of the majority consensus BI tree. The Infections/Infestation category of the organ targeted EBDCS (Figure 2A) classification method and the biological (i.e. antimicrobial response) method (Figure 2B) were plotted on the BI tree.

**FIGURE 3:**
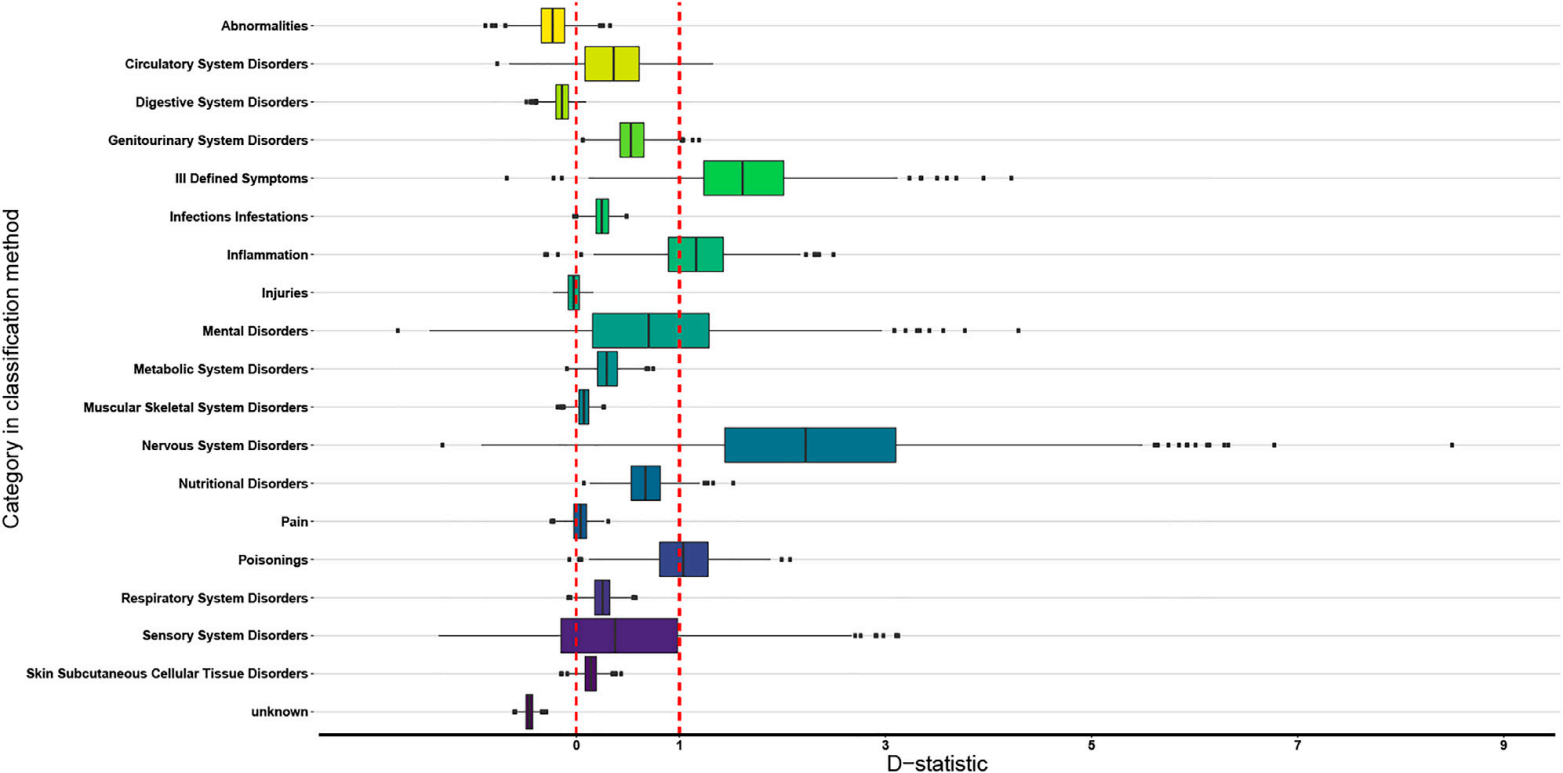
Boxplot of the 19 categories of the organ targeted EBDCS classification method (indicated with different colors) over which the data on medicinally used necklace orchid species that were analysed phylogenetically can be divided. The red lines indicate the D reference values 0 (on the left) and 1 (on the right). The box boundaries indicate the first and third quartile (Q1 and Q3), the line indicates the median, and the whiskers extend to either the extreme values or 1.5 times the interquartile range (Q3–Q1).

**FIGURE 4:**
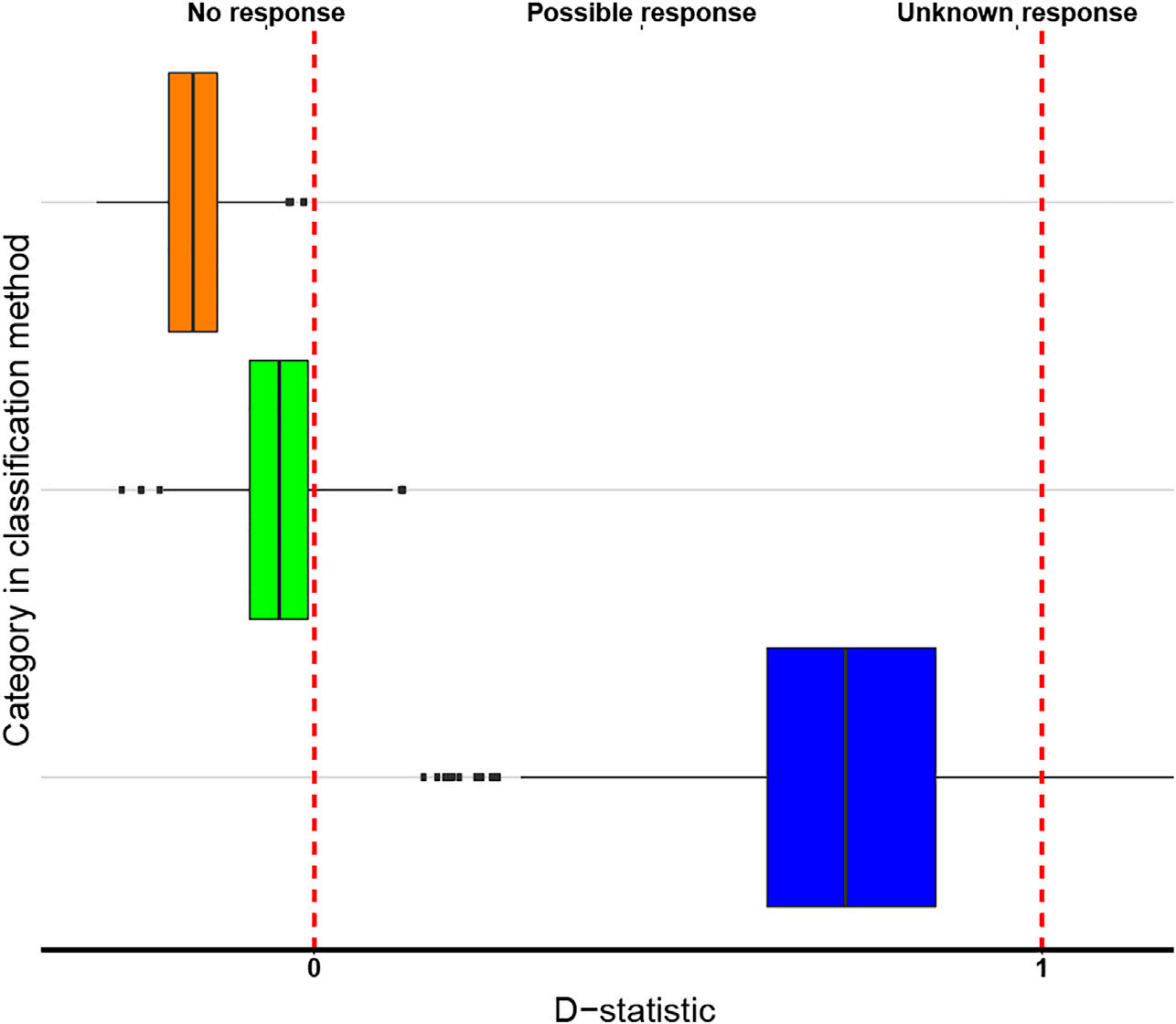
Boxplot of the Antimicrobial biological response classification method of the three characters states over which the data on medicinally used necklace orchid species, that were analysed phylogenetically, could be divided. Color codes: no response (orange), possible response (green), unknown response (blue). The red lines indicate the D reference values 0 (on the left) and 1 (on the right). The box boundaries indicate the first and third quartile (Q1 and Q3), the line indicates the median, and the whiskers extend to either the extreme values or 1.5 times the interquartile range (Q3–Q1).

The boxplots of the D-statistics for the organ-based EBDCS classification method and the antimicrobial biological response classification method are shown in Figures 3 and 4. For the EBDCS classification method, 7 of the 19 categories showed a D > 1, indicating that a minority of these categories were randomly distributed. A total of 12 of the 19 categories were (extremely) clustered. For the antimicrobial response method, all the categories were found to be (extremely) clustered.

**FIGURE 5:**
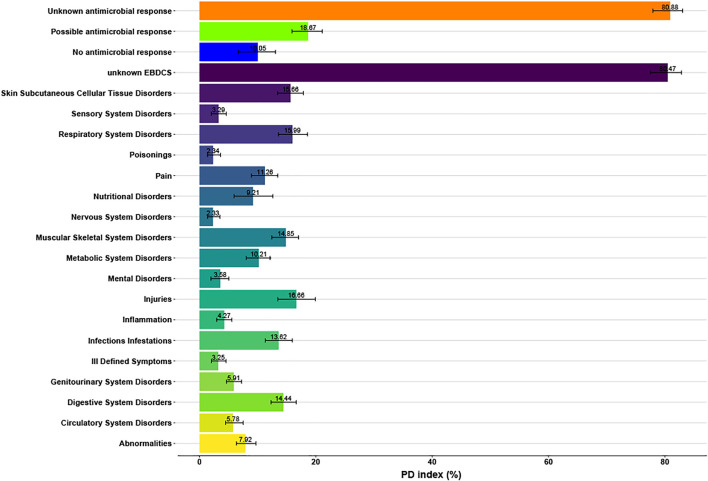
Median and standard errors of the Phylogenetic Diversity (PD) indices (in %) of the biological (i.e. antimicrobial) response and organ targeted EBDCS classification methods (indicated with different colors).

The median of the phylogenetic diversity (PD) was calculated to compare the phylogenetic distribution of medicinal species from the Unknown, Possible and No Antimicrobial Response categories with the 19 categories of the organ based EBDSC classification method. In Figure 5, these medians are depicted. The Possible Antimicrobial Response category of the biological classification method had a median of 18.83%, whereas the Infections/Infestations category of the organ based EBDSC classification method had a median of 13.32%.

To narrow down potential new species with antimicrobial activities, the Possible Antimicrobial Response state of the biological response classification method was compared with the Infections/Infestations category of the EBDCS classification method using the PHYLOCOM platform. Figure 2 depicts the recovered hot nodes. Figure 2A shows the three hot nodes detected for the category within the EBDCS classification method with high potency for antimicrobial activities. Figure 2B shows the eight hot nodes detected for the biological response classification method.

## Discussion

When compiling data on medicinal use of necklace orchids from the scientific literature, we noted that information for specific species was not always provided. This was for instance the case for the genus *Dendrochilum*. We thus urge ethnobotanists to make vouchers so that more detailed information for a particular genus can be obtained to link medicinal uses to the species level. Detailed information on plant organs used for medicinal purposes was not always provided either. We therefore recommend ethnobotanists to ask more detailed questions about specific organs used when interviewing traditional plant healers working with orchids. When information about plant organs was mentioned, this was sometimes contradictory among different publications. Our bio-assays showed that antimicrobial effects for extracts of leaves from *C. fimbriata* were much higher than those for pseudobulbs, which is not fully in agreement with previous publications, where it was found that pseudobulbs were the main source of secondary metabolites (Tóth et al., 2018). Our results are however supported by the publication of Buyun et al. (2016), who found that leaf extracts from *Coelogyne ovalis* produced larger inhibition zones than pseudobulbs extracts. The ethanol extract of leaves for *Bulbophyllum neilgherrense* also showed the same result (Priya and Krishnaveni, 2005). A possible explanation might be that the metabolites present in the pseudobulbs are more diluted as the relative amount of water is usually higher in these organs than in the leaves.

The exact method used to obtain plant extracts was also not always provided. Extracts dissolved in 70% ethanol had a higher antimicrobial effect in our bio-assays than extracts dissolved in hexane. This difference might be explained by the fact that hexane is a non-polar solvent that mostly extracts large fatty acid methyl esters with hydrocarbons and terpenes, whereas known antimicrobial substances isolated from necklace orchids are mostly phenanthrenes (Majumder et al., 2001; Kovács et al., 2008; Yang et al., 2012; Pant, 2014; Qian et al., 2015), which dissolve more readily in polar solvents such as ethanol.

Whether medicinal orchids were collected in the wild or from gardens or greenhouses was not mentioned in any of the publications that we screened. Our bio-assays show that antimicrobial effects of extracts of plants grown outside were higher (but not significantly so) than those of plants cultivated in greenhouses. A possible explanation for the difference in antimicrobial activity may be that plants naturally produce secondary metabolites that have a role in the defence against abiotic and biotic stresses (Dangle and Jones, 2001; Kim et al., 2009; Ramakrishna and Ravishankar, 2011). Recent studies by Isah (2019) also show that both stress and defense responses are involved in secondary metabolite production in plants. The insignificant differences found in our experiments when comparing indoor vs. outdoor cultivation methods might be a result of a too short exposure to UV light and herbivory, resulting in a too low level of secondary metabolites to create a significant difference between the cultivation methods. Plants grown in temperature controlled sterile greenhouses are generally exposed to less abiotic (UV light) and biotic (herbivores) stress and might therefore produce fewer secondary metabolites. Li et al. (1996) for instance reported that a longer exposure period to direct sunlight promoted higher ginsenoside production in American ginseng plants. Nevertheless, our results show that while exposure to UV light and herbivores may increase the antimicrobial activity of leaf extracts for necklace orchids, plants grown indoors also possess antimicrobial activity. This result contradicts the common folk belief that medicinal orchids can only be harvested from the wild to maintain their potency. We therefore encourage cultivation of medicinal necklace orchids in order to prevent overexploitation and extinction of rare species in the wild.

The 70% ethanol leaf extracts of *C. fimbriata* showed *in vitro* antimicrobial activity against *S. aureus, B. cereus* and *Y. enterocolitica,* all known to cause gastrointestinal tract infections in humans. Activity was observed against both Gram-positive and Gram-negative bacteria, which indicates a broad spectrum of antimicrobial effects of leaf extracts of necklace orchids. The extracts were not able to inhibit growth of *E. coli* and *K. pneumoniae*. This can be explained by the fact that Gram-negative bacteria generally develop more resistance against synthetic antibiotics as compared with Gram-positive bacteria because they can more efficiently regulate genes involved in antibiotic drug resistance (Peleg and Hooper, 2010).

In contrast with the organ targeted EBDCS classification method, all the categories from the biological response (i.e. antimicrobial) classification method were found to be (highly) clustered. The biological response classification method can therefore be considered as more informative for bioprospecting. The biological response classification method also had a more scattered distribution of medicinal species on the phylogeny than the EBDCS classification method, covering a wider group of potential medicinal necklace orchid species by retrieving eight hot nodes as compared with the organ targeted EBDCS classification method, that only found three hot nodes. One of the eight hot nodes detected by the biological response classification method, but not by the organ targeted EBDCS classification method, encompasses species of the necklace orchid genus *Bletilla*. Yang et al. (2012) successfully isolated bletilin A, bletilin B and other phenanthrenes from *Bletilla ochracea* tuber extracts that showed antibacterial activities against *S. aureus*, *S. epidermis* and *B. subtilis.* The fibrous roots and tubers from *Bletilla striata* contain biphenanthrenes and stilbenoids, which possess antibacterial activity (Kovács et al., 2008; Qian et al., 2015), Additionally, dihydrophenanthrenes, phenanthrene, flavonoids, bibenzyl and phenolic compounds were isolated from entire plants of *B. formosana* by Lin et al. (2005). These research findings support the results of our bioprospecting analyses and show that the biological response classification method is more effective in uncovering potential clades with high medicinal potential as compared with the EBDCS classification method.

Ethno-directed approaches to identifying plants traditionally used to treat specific diseases received significantly more attention over the past decade as this method shows a relatively high success rate compared to random plant screening programmes (Douwes et al., 2008; Siqueira et al., 2012). Plotting ethno-pharmacological data on a phylogenetic tree can be used as a time-efficient approach to discover potential new plant species with medicinal properties (Ernst et al., 2015), especially when a plant group is as large and diverse as the orchid family. We could only analyse 10% of all necklace orchid species for their medicinal properties. The reason for this was that while for some species with recorded medicinal use no DNA sequences were available, other species with known DNA sequences had not yet been investigated for their medicinal uses. We encourage more work into the ethnobotany and pharmacology of necklace orchids to increase species sampling. Especially species of the genera *Bletilla*, *Coelogyne* sect. *Bicellae*, sect. *Brachypterae*, sect. *Coelogyne,* sect. *Elatae,* sect. *Flaccidae,* sect. *Fuscescentes,* sect. *Hologyne,* sect. *Lawrenceana,* sect. *Lentiginosae*, sect. *Longifoliae,* sect. *Ocellatae,* sect. *Proliferae,* sect. *Ptychogyne,* sect. *Speciosae*, *Neogyna, Otochilus* and *Pholidota* sect. *Articulatae*, sect. *Chinenses*, sect. *Crinonia*, sect. *Pholidota* and sect. *Repentes* seem very promising for further research as these were identified to belong to hot node clades with high potency of antimicrobial activity.

## Conclusion

We successfully applied bioprospecting to discover new necklace orchid species with antimicrobial activity. The traditional antimicrobial use of necklace orchids could be confirmed with bio-assays for leaf extracts prepared with 70% ethanol. Additionally, outdoor cultivation may result in increased antimicrobial activity, though this needs to be further explored. The biological response classification method was more effective in uncovering hot nodes leading to clades of species of necklace orchids with high antimicrobial potential as compared to the EBDCS classification method.

## Data Availability

The datasets presented in this study can be found in online repositories. The names of the repository/repositories and accession number(s) can be found in the article/Supplementary Material.
